# Pregnancy-Specific Beta-1-Glycoprotein: The Player of the Game in predicting adverse outcomes in pregnancy

**DOI:** 10.5935/1518-0557.20220077

**Published:** 2023

**Authors:** Kenan Toprak, Rahime Kada

**Affiliations:** 1 Department of Cardiology, Siverek State Hospital, Sanliurfa, Turkey; 2 Obstetrics and Gynecology, Harran University Faculty of Medicine, Sanliurfa, Turkey

We read the recently published article by Tuzluoğlu *et al*. (2022) entitled ‘Investigation of Serum Pregnancy-Specific Beta-1-Glycoprotein and Relationship with Fetal Growth Restriction’ with great interest. In their well-designed and presented paper, the authors attempted to evaluate the relationship between Pregnancy-Specific Beta-1-Glycoprotein and fetal growth restriction. Although the relationship between Pregnancy-Specific Beta-1-Glycoprotein and fetal growth restriction was not statistically significant, they found a close relationship *(p*=0.058).

It is highly likely that increasing the number of subjects included in the study will make the results of the study meaningful. Because, in many previous studies, the fact that Pregnancy-Specific Beta-1-Glycoprotein is closely associated with some adverse pregnancy outcomes such as small-for-gestational age fetuses (SGA), spontaneous preterm delivery and preeclampsia supports our prediction (Pihl *et al*., 2009; Temur *et al*., 2020). As a matter of fact, in many previous clinical studies, adverse pregnancy conditions such as small-for-gestational age fetuses (SGA), spontaneous preterm delivery, and preeclampsia have been shown to be closely associated with fetal growth restriction (McCowan *et al*., 2018; Goldenberg *et al*., 2008; Obata *et al*., 2020).

However, the fact that some clinical and laboratory parameters were not evaluated between groups may have affected the results of the study. For example, basic hematological parameters such as fasting plasma glucose and lipid profile are not mentioned in the methodology. However, it has been shown that fasting blood glucose and maternal lipid profile are associated with fetal growth retardation in the absence of chronic disease (Guo *et al*., 2021; Alahakoon *et al*., 2020). Were these parameters evaluated between groups in the study?

In addition, are clinical features specific to pregnancy that may be associated with fetal growth retardation, such as gestational hypertension, gestational diabetes, and preeclampsia, have been recorded (Zhang *et al*., 2022; Bedell *et al*., 2021; Marasciulo *et al*., 2021)?

Finally, Pregnancy-specific β-glycoprotein 1 is synthesized by the human placenta and secreted into the maternal circulation in an increasing concentration from trophoblast differentiation to term (Zhou *et al.,* 1997). Although it is stated in the article that it is studied from blood samples in the last trimester, the last trimester corresponds to a period of 12 weeks. And this means that there is a possibility that blood samples may vary greatly in serum levels of Pregnancy-Specific Beta-1-Glycoprotein depending on the week taken. Is there data on which weeks blood samples were taken from the subjects? If yes, how does this affect the results of the research?

We believe that the evaluation of these parameters in the study will further increase the clinical importance of the study.
